# Genome evolution of SARS-CoV-2 and its virological characteristics

**DOI:** 10.1186/s41232-020-00126-7

**Published:** 2020-08-10

**Authors:** So Nakagawa, Takayuki Miyazawa

**Affiliations:** 1 Molecular Life Science, School of Medicine, 143 Shimokasuya, Isehara, Kanagawa 259-1193 Japan; 2 Institute of Medical Sciences, Tokai University, Kanagawa, Japan; 3 Micro/Nano Technology Center, Tokai University, Hiratsuka, Japan; 4 Laboratory of Virus-Host Coevolution, Institute for Frontier Life and Medical Sciences, 53 Shogoin-Kawaharacho, Sakyo-ku, Kyoto, 606-8507 Japan; 5 Resilience Research Unit, Kyoto University, Kyoto, Japan

**Keywords:** COVID-19, Coronavirus, Comparative genomics, SARS-CoV-2, Viral evolution

## Abstract

Coronavirus disease of 2019 (COVID-19), which originated in China in 2019, shows mild cold and pneumonia symptoms that can occasionally worsen and result in deaths. SARS-CoV-2 was reported to be the causative agent of the disease and was identified as being similar to SARS-CoV, a causative agent of SARS in 2003. In this review, we described the phylogeny of SARS-CoV-2, covering various related studies, in particular, focusing on viruses obtained from horseshoe bats and pangolins that belong to *Sarbecovirus*, a subgenus of *Betacoronavirus*. We also describe the virological characteristics of SARS-CoV-2 and compare them with other coronaviruses. More than 30,000 genome sequences of SARS-CoV-2 are available in the GISAID database as of May 28, 2020. Using the genome sequence data of closely related viruses, the genomic characteristics and evolution of SARS-CoV-2 were extensively studied. However, given the global prevalence of COVID-19 and the large number of associated deaths, further computational and experimental virological analyses are required to fully characterize SARS-CoV-2.

## Background

On December 12, 2019, an epidemic of acute respiratory syndrome in humans started in the city of Wuhan, Hubei province, central China [1–4]. The causative agent of the symptom was found to be a novel coronavirus (CoV), of which genome is phylogenetically similar to that of the severe acute respiratory syndrome (SARS) CoV (SARS-CoV) [1–4]. Because of that, World Health Organization (WHO) named the symptoms coronavirus disease 19 (COVID-19) [5], and the Coronaviridae Study Group of the International Committee on Taxonomy of Viruses (ICTV) named the novel CoV as SARS-CoV-2 [6]. In this review, we noted characteristics of SARS-CoV-2 compared to those of other CoVs.

## Main text

### Phylogeny of SARS-CoV-2

SARS-CoV-2 is a member of the coronavirus family (*Coronaviridae*). The family *Coronaviridae* is a relatively large family that includes a variety of viral species. The coronavirus family is divided into two subfamilies: *Letovirinae* and *Orthocoronavirinae* [7]. SARS-CoV-2 is classified as an orthocoronavirus subfamily member. The orthocoronavirus subfamily is further divided into four genera: *Alphacoronavirus*, *Betacoronavirus*, *Gammacoronavirus*, and *Deltacoronavirus* [7]. In addition, the genus *Betacoronavirus* is reported to be divided into five subgenera: *Sarbecovirus*, *Hibecovirus*, *Nobecovirus*, *Merbecovirus*, and *Embecovirus* [7, 8].

**Fig. 1:**
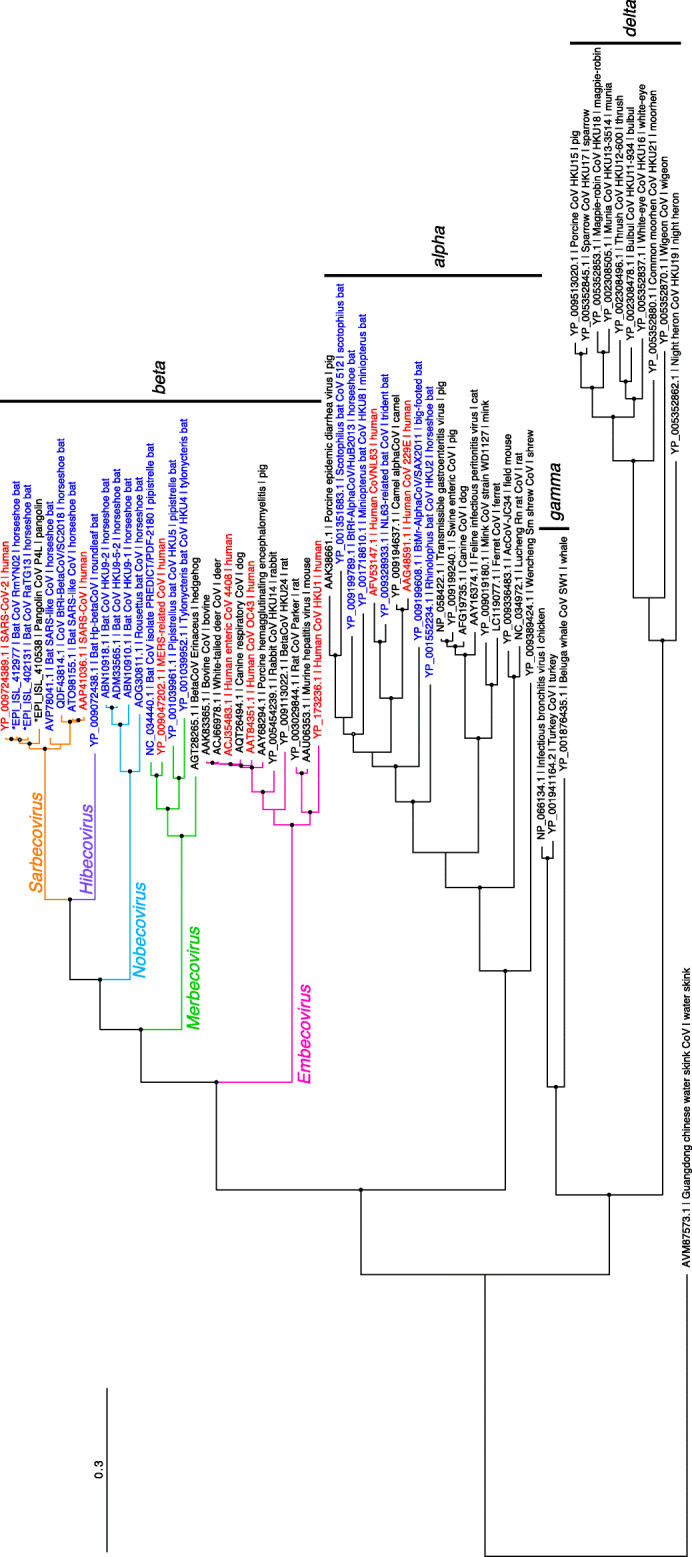
Phylogeny of orthocoronaviruses. Maximum likelihood (ML)-based phylogenetic tree of 61 orthocoronaviruses. Partial amino acid sequences of ORF1ab were used for the analysis. We generated the multiple alignment of the sequences using L-INS-i of MAFFT version 7.453 [9], and the amino acid substitution model LG+I+G was selected using ProtTest3 [10]. Based on the model, we constructed an ML tree using RAxML-NG [11] applying 1000 bootstrapping tests. GenBank or GISAID (that was indicated by asterisk (*)) accession number, strain name, and host of each virus are indicated for each branch terminal. CoVs obtained from humans or bats are shown in red or blue, respectively. A black or open circle corresponds to bootstrap values ≥ 95% or ≥ 80%, respectively. The scale is shown in the upper left

The maximum likelihood (ML) tree based on amino acid sequences of open reading frame 1ab (ORF1ab) indicated the phylogenetic relationship of various CoVs shown in Fig. 1. The phylogenetic tree was constructed from 61 viruses belonging to the orthocoronavirus subfamily. More than 100 CoVs were isolated from various mammalian and avian species, and the CoVs shown in Fig. 1 are representatives selected by the authors to illustrate diversity of CoVs, of which complete genomes are available in public databases excluding an unclassified coronavirus found in *Tropidophorus sinicus* (Chinese waterside skink). The Guangdong Chinese water skink CoV was used as an outgroup in Fig. 1, which was the only CoV found in reptiles other than mammals and birds [12]. SARS-CoV-2, along with SARS-CoV and Middle East respiratory syndrome (MERS)-CoV, is classified in the genus *Betacoronavirus*. SARS-CoV-2 and SARS-CoV belong to the subgenus *Sarbecovirus*, accompanying various CoVs found in bats, in particular from horseshoe bats (genus *Rhinolophus*).

In addition to SARS-CoV-2, SARS-CoV, and MERS-CoV, there are four other CoVs that cause common cold symptoms in humans: human CoV (HCoV) HKU1 and HCoV OC43, belonging to the genus *Betacoronavirus*, and HCoV 229E and HCoV NL63, belonging to the *Alphacoronavirus*. Although there are few reported cases, human enteric coronaviruses (HECV) that cause diarrhea in humans belong to the *Betacoronavirus* genus. Viruses closely related to HCoV HKU1 are present in rodents, and HECV is closely related to CoVs isolated from even-toed animals (bovine and deer). These data indicate that these HCoVs were derived from CoVs of domestic animals and small animals such as rodents. There are multiple types of CoVs in non-human animals, and it is undeniable that coronaviral transmissions from domestic, companion, and wild animals to humans might have occurred many times without people realizing it.

**Fig. 2:**
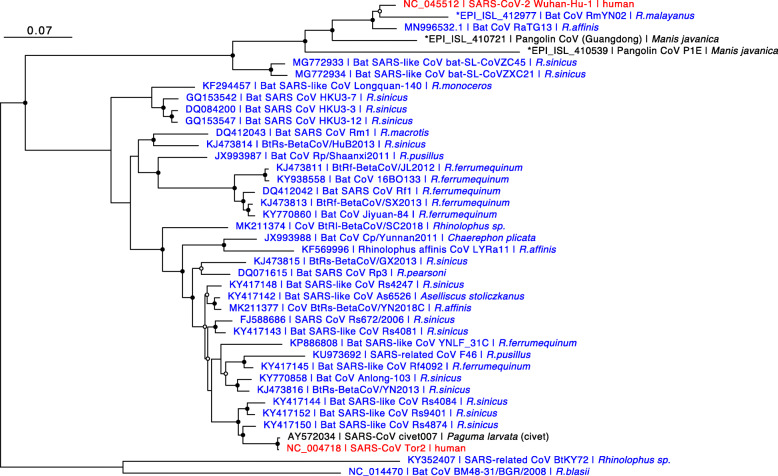
Phylogeny of CoVs belonging to *Sarbecoronavirus*. ML-based phylogenetic tree of 41 CoVs belonging to *Sarbecoronavirus* including SARS-CoV-2. Whole genome sequences were used for the analysis. We generated the multiple alignment of the sequences using L-INS-i of MAFFT version 7.453 [9], and the nucleotide substitution model GTR+I+G was selected using ModelTest-NG [13]. Based on the model, we constructed an ML tree using RAxML-NG [11], applying 1000 bootstrapping tests. Please see Fig. 1 legend for the details of this figure

The phylogenetic relationship of SARS-CoV-2 with other closely related CoVs belonging to subgenus *Sarbecovirus* is illustrated in Fig. 2. Note that entire genomic sequences were used for this phylogenetic analysis. CoVs which are the most closely related to the SARS-CoV-2 are Bat CoVs, in particular strains RmYN02 [14] and RaTG13 [4], both of which are isolated from horseshoe bats (genus *Rhinolophus*). Further, CoVs found in Malaysian pangolins are the next closest to SARS-CoV-2 as well. These observations are also indicated by Fig. 1, which is based on partial amino acid sequences of the ORF1ab gene. As shown in the Fig. 2, most of the CoVs belonging to subgenus *Sarbecovirus* were found in horseshoe bats or other bat species. Therefore, although we still do not know the direct origin of SARS-CoV-2, it is highly possible that CoV(s) belonging to *Sarbecovirus* in horseshoe bats could be the origin of SARS-CoV-2.

### Phenotypic features and genomic structures of SARS-CoV-2

The phenotypic features of CoVs are as follows. The viral particles are spherical, 100 to 120 nm in diameter, with envelopes derived from the host cell membrane. CoVs were named “coronaviruses” because they are characterized by spike protein projections on the surface of the viral particles (about 20 nm in length), and their shape resembles a crown (corona) under electron microscopy. Those features are embodied in SARS-CoV-2 [1].

**Fig. 3:**
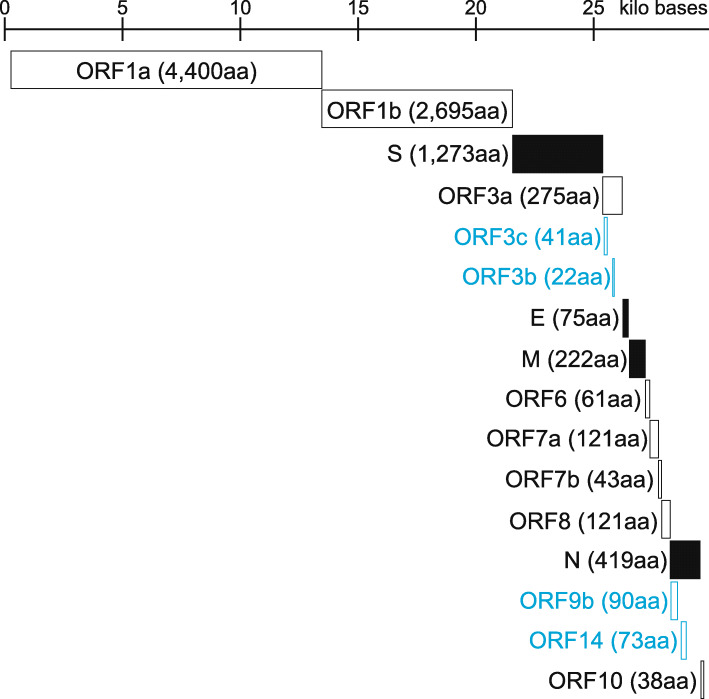
Genomic structure of SARS-CoV-2. Schematic genomic structure of SARS-CoV-2 was shown based on the SARS-CoV-2 Wuhan-Hu-1 (NCBI Reference Sequence ID: NC_045512.2). The scale was shown on the top. Each ORF was illustrated based on the NCBI annotation of NC_045512.2, and a rectangle filled with black corresponds to a structural protein. The number in parentheses is the length of amino acid sequence (aa, amino acid). A gene name as well as rectangle colored in light blue was a hypothetical ORF which is not annotated NC_045512.2 currently. ORF3b is based on Konno et al. [15], and the others are based on Davidson et al. [16] and Jungreis et al. [17]

The genome structure of CoVs is a non-segmented, positive-sense single-stranded RNA (+ssRNA). The genome size ranges from 27 to 32 kb: a cap structure at the 5′ end followed by a reader sequence of about 70 bases, several ORFs coding various proteins, and a non-translated region including a poly-A sequence at the 3′ end. Figure 3 shows the genomic structure of SARS-CoV-2 (29.9 kb). For the ORFs from the 5′ end, a region of about 20 kb corresponds to the two ORFs (ORF1a and ORF1b). ORF1a and ORF1b encode 11 and 5 non-structural proteins: nsp1 to nsp11 and nsp12 to 16, respectively. ORF1a is translated directly from the genomic RNA; however, expression of ORF1b requires a − 1 ribosomal frameshift near the end of ORF1, resulting in a single ORF1ab polypeptide. Downstream from the ORF1ab, there are ORFs encoding a few to more than ten structural/non-structural proteins. The common structural proteins of CoV subfamily viruses are nucleocapsid (N), spike (S), membrane (M), and envelope (E) proteins. The S protein is responsible for both binding to receptors expressed on the cell membranes of susceptible cells and membrane fusion. The M and E proteins are involved in the assembly and budding of viral particles. CoVs also code various non-structural proteins in ORF1ab as well as in other ORFs, in particular near the 3′ end, although the details of the exact genes in the SARS-CoV-2 genome are still unclear mainly due to overlapping genes encoded in a different coding frame as illustrated in Fig. 3.

The SARS-CoV-2 genome shares nucleotide identity to the genomes of Bat CoV RaTG13 (96%) [4], Bat CoV RmYN02 (93%) [14], Pangolin CoV (90%) [18–20], SARS-CoV (80%) [4], and MERS-CoV belonging to *Merbecovirus* (50%) [21]. However, the nucleotide identity varied greatly depending on genes as well as genomic loci [4, 14, 18–22]. For example, the receptor-binding domain of S genes of SARS-CoV-2 is very similar to that of Pangolin CoVs, rather than those of Bat CoVs RaTG13 and RmYN02 [14, 18], while a polybasic (furin) cleavage site, which is one of the prominent features of SARS-CoV-2 [23, 24], was found only in Bat CoV RmYN02 among CoVs belonging to the subgenus *Sarbecovirus* [14]. ORF1ab of SARS-CoV-2 is quite similar to that of Bat CoV RmYN02 rather than that of RaTG13 [14]. Those complex genomic features could be a consequence of inter-viral recombination [25]. With respect to the differences in each gene of *Sarbecovirus*, it was reported that ORF3b differs greatly in length among viruses belonging to the *Sarbecovirus* genus, including SARS-CoV-2 and SARS-CoV, and that these differences could contribute to differences in the anti-interferon activity [15]. Moreover, it was found that there are SARS-CoV-2 variants showing a longer ORF3b, which were isolated from two patients with severe diseases [15]. This observation may indicate an increased the ability of the longer ORF3b to suppress interferon induction in those patients.

### Genome sequencing data analyses of SARS-CoV-2

SARS-CoV-2 information including genome sequencing data was collected in a database called GISAID (Global Initiative on Sharing All Influenza Data, https://www.gisaid.org) [26], which shares sequence data on potentially pandemic infectious viruses, as well as methods for sequencing and relevant geographic and clinical information. The GISAID database includes sequencing data that are not available in public nucleotide databases such as GenBank. As the name implies, this database was constructed at the time of the influenza A H1N1 2009 pandemic, but it covers SARS-CoV-2 in view of urgency. In the GISAID database, not only SARS-CoV-2 but also highly similar viral sequences such as CoVs isolated from bats and pangolins have been collected. Based on the viral sequences as well as geographical and sample collection information in the GISAID database, Nextstrain (https://nextstrain.org) [27] shares phylogenetic, geographical, and genomic analyses of SARS-CoV-2, illustrating the real-time evolution of SARS-CoV-2. Note that Nextstrain has been used to analyze the phylogeny of not only SARS-CoV-2 but also other pathogenic viruses that can potentially pose a public health threat. At the time of writing this article (May 28, 2020), 30,699 SARS-CoV-2 and closely related viral sequences are stored in the GISAID database, and 4308 SARS-CoV-2 genomes were analyzed in the Nextstrain. According to the Nextstrain, the number of substitutions in the SARS-CoV-2 genome was estimated at approximately 26 substitutions per year. Considering the size of SARS-CoV-2 genome (29.9 kb), the estimated evolutionary rate is approximately 0.90 × 10^−3^ substitution/site/year. The value of this evolutionary rate is similar when compared to other previously reported rates of SARS-CoV (0.80–2.38 × 10^−3^, Zhao et al.) [28], MERS-CoV (0.63–1.12 × 10^−3^) [29–31], and HCoV OC43 (0.43 × 10^−3^) [32]. To the best of our knowledge, the mutation rate (the number of substitutions per site per replication cycle) of SARS-CoV-2 has not been examined yet, but it could be lower than that other RNA viruses such as influenza viruses because the SARS-CoV-2 genome encodes a proofreading exoribonuclease called ExoN in nonstructural protein 14 (nsp14) of the ORF1b as it was reported in SARS-CoV [33].

We know that the evolution of coronaviruses occurs not only by nucleotide mutations but also by recombination. In particular, it has been suggested that the feline infectious peritonitis virus, which causes lethal infectious peritonitis in cats, was caused by recombination of a feline coronavirus with a canine coronavirus [34]. Furthermore, porcine infectious peritonitis virus transforms into porcine respiratory coronavirus (PRCV), which causes respiratory disease when a portion of the S protein is deficient [35]. In murine hepatitis virus (MHV), three amino acid mutations were found to be associated with demyelination and hepatitis [36].

No conclusions have been reached as to whether amino acid mutations are responsible for the difference in SARS-CoV-2 virulence, although certain nucleotide mutations are widely spread in the population. Tang et al. reported that the current coronavirus was divided into two genotypes (designated L and S) depending on an amino acid site 84 (S84L) of ORF8 gene [37]. When compared with closely related CoVs such as Bat CoV RaTG13 and Pangolin CoVs, the ancestral type of SARS-CoV-2 was thought to be S-type [37]. However, the L-type emerged in the beginning of the COVID-19 outbreak, and the current major type of SARS-CoV-2 widely spreading all over the world is L-type as of May 21, 2020 (https://nextstrain.org). Zhang et al. analyzed the clinical and immunological data from 326 confirmed cases of COVID-19 and compared them with viral genetic variation including the S84L mutation, but they could not find any association among them [38]. Korber et al. reported a mutation at an amino acid site 614 (D614G) of S protein that is currently dominant in Europe [39]. Since the S protein is essential in infecting cells and is a primary target for neutralizing antibodies, the mutations in the S protein could be related to the virulence; however, this hypothesis should be evaluated experimentally using reverse genetics. Although more than 5000 mutations accumulated in the SARS-CoV-2 population [40], there are no shreds of evidence currently supporting that SARS-CoV-2 genomes are separating into distinct genotypes during the evolution [41].

## Conclusion

Although only about a half year has passed since a genome sequence of SARS-CoV-2 was shared in the GISAID database, more than 30,000 genomes are now available. Using the genome sequence data with closely related viral genome data, the genomic characteristics and evolution of SARS-CoV-2 were extensively studied. However, SARS-CoV-2 is still prevailing around the world and is causing many deaths. Further viral genomic and experimental virological analyses are required to characterize SARS-CoV-2.

## Data Availability

Phylogenetic data shown in this review are available upon request.

## Abbreviations

aa: Amino acid
CoV: Coronavirus
COVID-19: Coronavirus disease 19
E: Envelope
HCoV: Human coronavirus
HECV: Human enteric coronaviruses
ICTV: International Committee on Taxonomy of Viruses
M: Membrane
ML: Maximum likelihood
MERS: Middle East respiratory syndrome
N: Nucleocapsid
ORF: Open reading frame
S: Spike
SARS: Severe acute respiratory syndrome
WHO: World Health Organization
