# Correction to: Genome evolution of SARS-CoV-2 and its virological characteristics

**DOI:** 10.1186/s41232-020-00151-6

**Published:** 2020-12-21

**Authors:** So Nakagawa, Takayuki Miyazawa

**Affiliations:** 1 Molecular Life Science, School of Medicine, 143 Shimokasuya, Isehara, Kanagawa, 259-1193 Japan; 2 Institute of Medical Sciences, Tokai University, Kanagawa, Japan; 3 Micro/Nano Technology Center, Tokai University, Hiratsuka, Japan; 4 Laboratory of Virus-Host Coevolution, Institute for Frontier Life and Medical Sciences, 53 Shogoin-Kawaharacho, Sakyo-ku, Kyoto, 606-8507 Japan; 5 Resilience Research Unit, Kyoto University, Kyoto, Japan

**Correction to: Inflamm Regener 40, 17 (2020)**

**https://doi.org/10.1186/s41232-020-00126-7**

After publication of the original article [1] the authors spotted an error in the last sentence of the 1st paragraph of “Phylogeny of SARS-CoV-2” in the Main text. This sentence originally stated:

- In addition, the genus *Betacoronavirus* is reported to be divided into four lineages (subgenera): Lineage A (subgenus *Embecovirus*), Lineage B (subgenus *Sarbecovirus*), Lineage C (subgenus *Merbecovirus*), and Lineage D (subgenus *Nobecovirus*) [7, 8].

However, Bat Hp-betaCoV belong to *Hibecovirus*. Therefore, the corrected sentence reads as followed:

- In addition, the genus *Betacoronavirus* is reported to be divided into five subgenera: *Sarbecovirus*, *Hibecovirus*, *Nobecovirus*, *Merbecovirus*, and *Embecovirus* [7, 8].

This error also affected Figure 1. The incorrect (Fig. 1) and correct (Fig. 2) version of figure 1 are shown in this correction article. The original article has been updated to rectify the above errors.

**Fig. 1:**
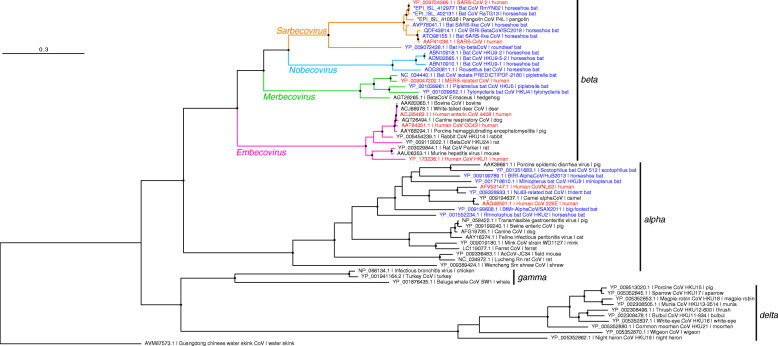
The incorrect version of figure 1 as originally published

**Fig. 2:**
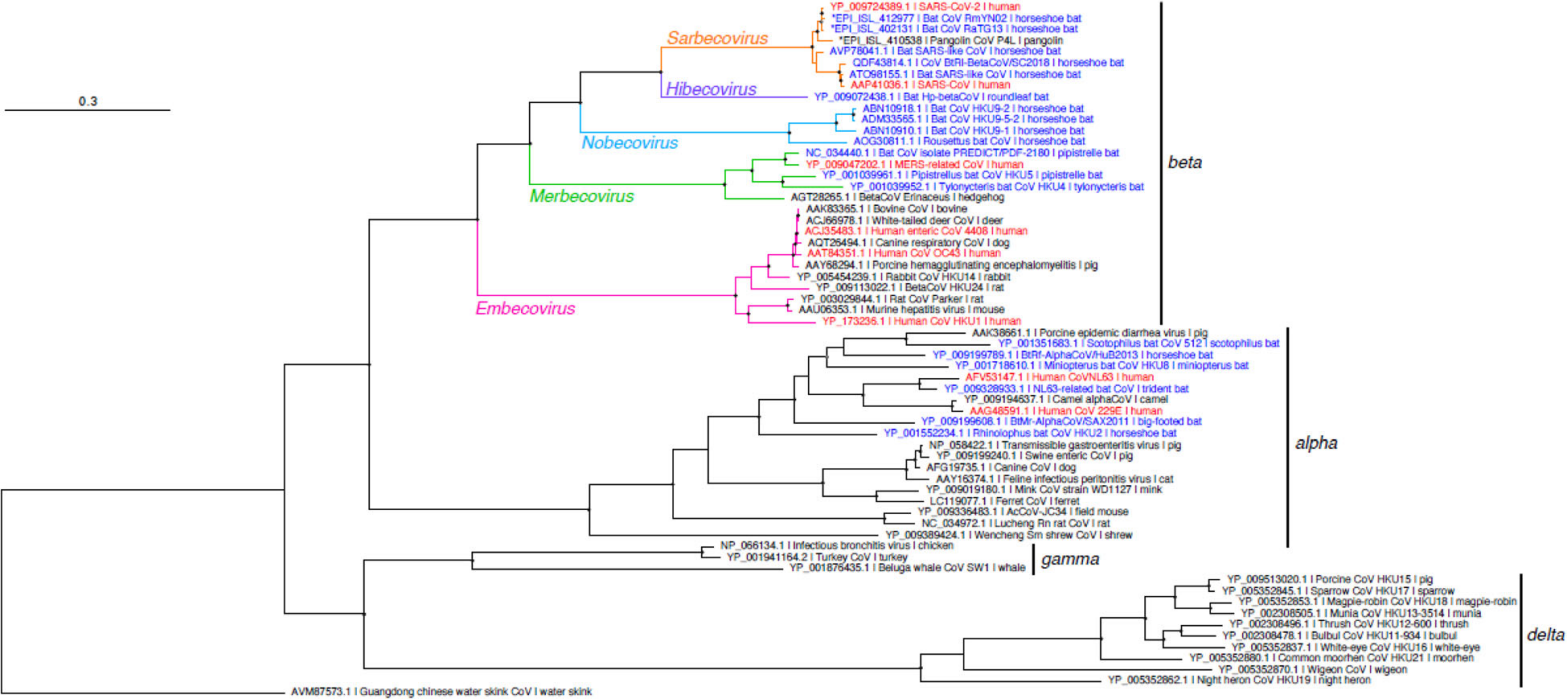
The correct version of figure 1
